# Influence of povidone-iodine on micro-tensile bonding strength to dentin under simulated pulpal pressure

**DOI:** 10.1186/s12903-018-0645-9

**Published:** 2018-10-29

**Authors:** Najlaa M. Alamoudi, Alaa M. Baik, Azza A. El-Housseiny, Tariq S. Abu Haimed, Ahmed S. Bakry

**Affiliations:** 1 Pediatric Dentistry Department, Faculty of Dentistry, King Abdulaziz University, P.O. Box 80209, Jeddah, 21589 Saudi Arabia; 2 Pediatric Dentistry Department, King Abdulaziz University Dental Hospital, Jeddah, Saudi Arabia; 3 Pediatric Dentistry Department, Faculty of Dentistry, Alexandria University, Alexandria, Egypt; 4 Biomaterial Department, Faculty of Dentistry, King Abdulaziz University, Jeddah, Saudi Arabia; 5 Operative Dentistry Department, Faculty of Dentistry, King Abdulaziz University, Jeddah, Saudi Arabia; 6 Conservative Dentistry Department, Faculty of Dentistry, Alexandria University, Alexandria, Egypt

**Keywords:** Matrix metalloproteinase, Micro-tensile bond strength, Povidone-iodine, Chlorhexidine

## Abstract

**Background:**

Previous studies had reported that bond strength deteriorate over time following the dentin surface pretreatment with chlorhexidine. Therefore, further investigations are needed to evaluate the effect of other materials such as povidone iodine.

The purpose of this study was to investigate the effects of 10% povidone-iodine pretreatment on the resin-dentin micro-tensile bond strength of a single bond adhesive system in permanent teeth over time, and compare it with 2% chlorhexidine.

**Methods:**

Flat dentin surfaces were prepared in 63 extracted permanent teeth. Teeth were randomly assigned to a 10% povidone-iodine pretreatment, a 2% chlorhexidine pretreatment, or a control group. Composite resin blocks were built up over treated surfaces under pulp pressure simulation. The prepared specimens were assigned to three storage time, 24 h, 1 week, and 2 months. Samples were vertically sectioned to obtain specimens of 0.7 to 1.2 mm^2^ cross-sectional area.

**Results:**

No significant reduction of bond strength of povidone iodine group was found among the three storage times (*p* = 0.477). A significant reduction of bond strength for both chlorhexidine and control groups was found in the three storage times (*p* <  0.001).

**Conclusion:**

Povidone iodine pretreatment of etched dentin was effective in reducing the loss of bond strength over time, while the chlorhexidine pretreatment and negative control showed significant deterioration in micro-tensile bond strength over time in permanent teeth.

## Background

A current area of interest in adhesive dentistry is the durability of resin restorations [1]. The generation of resin-dentin bonds entails various challenges, including the preservation of structural integrity and strength. The bonds between resin and dentin using dentin adhesive systems can become damaged over time [2]. Aged composite resin bonded to dentin reportedly exhibited hydrolytic degeneration of collagen [1] without any bacterial enzymes [2].

Matrix metalloproteinases (MMPs) are part of the composition of dentin structure [3]. They are a group of proteases that can destroy the organic matrix of etched dentin [4]. All MMP family members are secreted as inactive proenzymes (Pro-MMPs). Pro-MMPs can be activated by proteases, other members of the MMP family, acids, reactive oxygen, and denaturants [2]. These Pro-MMPs need calcium ion (Ca^2+^) for their activation. Following bacterial demineralization, Ca^2+^ ions are released and act as a cofactor for the Pro-MMPs activation. These MMPs lead to dentin destruction. Similarly, following acid etching demineralization, MMPs are released and activated [2].

Establishing adhesion to mineralized tissue is based on the reaction of biologic apatite with acids. Etching dentin with 37% phosphoric acid lead to hydroxyapatite crystals dissolution, demineralization of the surface of the dentin matrix, exposure of the underlying collagen and creates porosities within that collagen matrix. A demineralized microporous area composed of organic dentin material mainly collagen fibrils is formed. This allows solvated monomers to infiltrate around and into spaces of collagen fibrils to obtain retention for resin-composite materials. However, acid etchings release the Ca^2+^ ions and activate MMPs. These MMPs lead to collagen destruction leading to enzymatic degradation of hybrid layer and subsequently endanger resin-dentin bonding longevity [4]. In order to prevent enzymatic degradation, inhibitors of MMP activity have been applied during adhesive application [5]. One such enzymatic inhibitor is chlorhexidine [2].

Povidone-iodine (PVP-I) was introduced as an antiseptic agent in the 1950s, and is as effective as iodine alone against a broad spectrum of disease-causing microorganisms [6, 7]. It is an organic water-soluble complex that contains molecular iodine and the solubilizing agent polyvinyl pyrrolidone. It is less irritating to the skin than iodine, and unlike iodine it does not require iodides or alcohol to dissolve. Moreover, PVP-I stains are water-soluble.

It has been claimed that PVP-I can induce an endogenous proteinase inhibitor capable of blocking enzymatic activity [8, 9]. Reductions in the collagenolytic activity of MMP-9 by 83% and MMP-2 by 88% were reported after 24 h of PVP-I application on nitrogen and sulfur mustard-induced skin lesions. When skin was analyzed 48 and 72 h after exposure, a similar trend of PVP-I-induced reduction in the two types of collagenase activity was found [8].

The hydrolytic and enzymatic stability of dental adhesives in the oral environment is a concern because the oral environment can severely compromise the durability of resin-dentin bonds. A literature review was performed prior to the current study, to investigate the effects of using chlorhexidine as a MMP inhibitor on dentin substrates, and the influence of chlorhexidine application on the preservation of dentin bond durability. Previous studies have reported that bond strength deteriorated over time following dentin surface pretreatment with chlorhexidine [10, 11]. This suggested that further investigations were needed to evaluate the effects of other materials such as PVP-I on the durability of dentin micro-tensile bond strength (MTBS).

The aim of this study was to evaluate the effects of 10% PVP-I pretreatment on dentin MTBS, and compare it with 2% chlorhexidine pretreatment and no pretreatment in permanent teeth over a period of 2 months.

## Methods

### Specimen preparation

The protocol of this study was approved by the ethics committee, King Abdulaziz University, Jeddah, Saudi Arabia. This is a randomized experimental in vitro study. Collecting bottles containing 0.5% Chloramine T were distributed to different private and governmental clinics in Jeddah to collect the freshly extracted sound teeth on it. Contact information was given to the dental assistant in charge for each clinic to contact us as soon as the teeth are extracted. Informed consent form was obtained and signed from patients to use their teeth for this research. Sixty-three extracted intact, non-carious, non-restored human permanent third molars were collected. The molars were mounted in 2-cm-diameter cylindrical customized molds using chemically cured acrylic resin (Major Prodotti Dentari Spa, Moncalieri, Italy). The roots were embedded 3 mm below the cementoenamel junction with the long axis of each tooth parallel to the walls of the mold. Each tooth was transversally cut in two steps using a slow speed diamond saw (TECHCUT 4™, Allied High Tech Products, Inc., USA) under copious water irrigation. The first cut was perpendicular to the long axis of each tooth at the coronal part of the crown, to remove the occlusal surfaces of the crowns leaving flat mid-coronal dentin. The subsequent cut was at a level approximately 1 mm below the cementoenamel junction and parallel to the flat dentin surface, to remove the root portion and expose the pulp chamber, from which the pulp tissue was carefully removed using tweezers.

### Simulating pulpal pressure

**Fig. 1:**
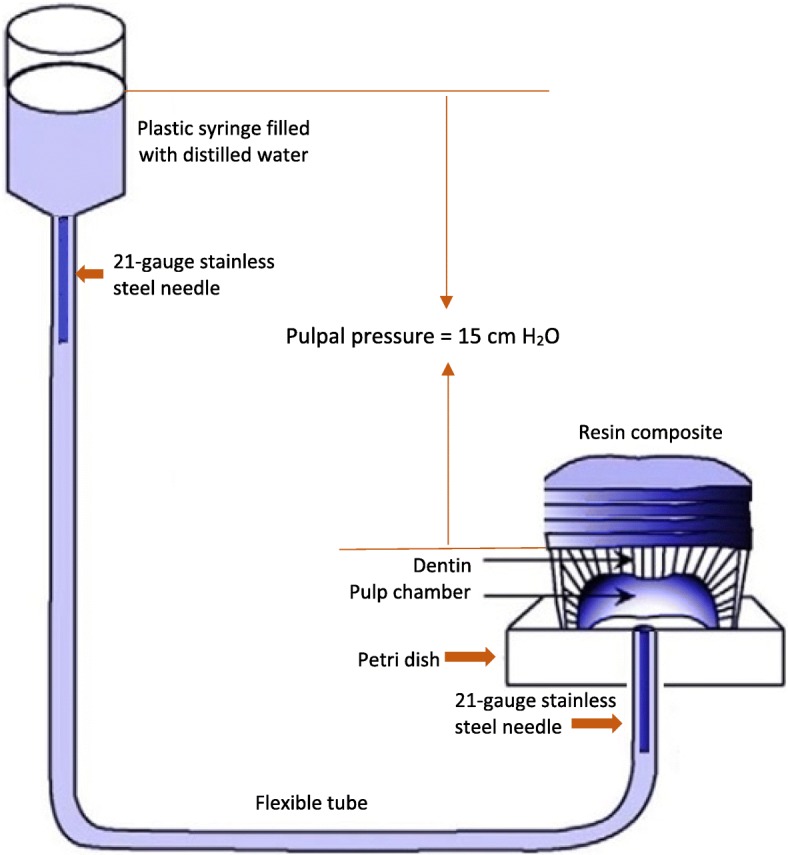
Illustration depicting the intra-pulpal pressure simulation apparatus used in this study

In an attempt to simulate intra-oral conditions, simulated pulp pressure was employed (Fig. 1). All restorative procedures including dentin adhesive bonding were performed under simulated dentinal hydrostatic pressure. Each segment was attached to a petri dish from the pulpal area and penetrated by a 21-gauge stainless steel needle. A flexible tube was connected and sealed to the stainless steel needle and to the back of the petri dish using cyanoacrylate adhesive (Zapit; DVA, Corona, CA, USA). The other end of this tube was sealed to a plastic syringe to maintain an airtight seal. The plastic syringe and the flexible tube were then filled with distilled water. The 1-mm root of the crown segment was bonded and sealed to the inner side of the petri dish using a cyanoacrylate adhesive. The intra pulpal pressure assembly was fixed to a burette stand. To generate an intra-pulpal pressure of 15 cm H_2_O, the level of distilled water in the 10-mL plastic syringe was adjusted 15 cm vertically above the flat dentin surface of the tested tooth.

### Acid etching and pretreatment

The teeth were randomly assigned to three groups according to the pretreatment solution. Each group had 21 teeth. The teeth were etched with 35% phosphoric acid gel (Scotchbond Universal Etchant, 3 M ESPE, St Paul, MN, USA) for 15 s and rinsed with water for 10 s. They were then placed in 10% PVP-I solution for 60 s in group 1, 2% chlorhexidine solution for 60 s in group 2, and in water in the control group (no pretreatment). The dentin surface was dried for 10 s with an oil-and-water-free air source with pressure to remove excess water and pretreatment solutions. Two coats of the single bond (Adper Single Bond 2, 3 M ESPE, St Paul, MN, USA) were applied to the entire flat occlusal surface of the dentin with 5 s of air-drying after each application, before being cured for 10 s with a quartz-tungsten-halogen light-curing unit (Curing Light 2500, 3 M EPSE) delivering 600 mW/cm^2^. A composite resin (Filtek Z250, 3 M ESPE, St Paul, MN, USA) crown of 4 mm in height was incrementally applied to the bonded dentin surface in 1-mm increments. A celluloid strip was used to separate the composite and the light curer tip. Each composite increment was light-cured for 30 s.

The prepared specimens were randomly assigned to three storage time subgroups containing 7 teeth each: 24 h, 1 week, and 2 months. Samples were stored in distilled water and placed in an incubator at 37 °C, and the distilled water was changed periodically.

### Sample preparation for MTBS

At each designated time-point (24 h, 1 week, 2 months), the teeth in the relevant subgroups were vertically sectioned across the bonded interface (in the occluso-gingival direction) into multiple serial sections using a low speed saw (TECHCUT 4™, Allied High Tech Products, Inc., USA) at a cutting speed of 200 rpm. Each crown segment was sectioned into two or three slabs, then each slab was rotated 90 degrees in the same plane and another two or three sections were made perpendicular to the slabs, resulted in four to six sticks. The cross-sectional areas of the selected specimens were measured using a digital caliper to the nearest 0.01 mm for subsequent bond strength evaluation.

### MTBS

To test MTBS, the end of each stick was fixed to the flat stainless steel microgrip of a universal testing machine (Micro-tensile Tester; Bisco, Schaumburg, IL) using cyanoacrylate glue (Zapit; DVA, Corona, CA, USA). The specimens were tension-stressed until failure occurred, using a simplified universal testing machine at a crosshead speed of 1 mm/minute. MTBS was calculated as the maximum load at failure divided by the cross-sectional area of each stick, and was recorded in MPa. The mean MTBS for each group was calculated and the three groups were compared.

### Failure mode analysis

The fractured specimens were kept in distilled water for 24 h. All debonded surfaces (dentin and resin) were evaluated under a stereomicroscope (Meiji Techno Co. Ltd., Tokyo, Japan) at 50x magnification to assess the mode of failure. The failure modes of the fractured specimens were categorized as adhesive failure when the fracture occurred between adhesive and dentin, cohesive failure in dentin when dentin covered the two fractured parts of the specimen, cohesive failure in resin material when the two parts of the specimens were fully covered with composite resin, or mixed failure when there were two or more of the types of fractures described above.

### Statistical analysis

The data were tested for normality using the Shapiro-Wilks test. One-way analysis of variance was conducted to assess the pretreatment effect on resin-dentin bond strength at each storage time-point, and to evaluate the effect of storage time on the resin-dentin bond strength within each pretreatment group (i.e., assuming the samples were independent). The post-hoc Bonferroni test was used for multiple pairwise comparisons to identify any significant differences between groups. All tests employed a 0.05 level of statistical significance.

## Results

**Fig. 2:**
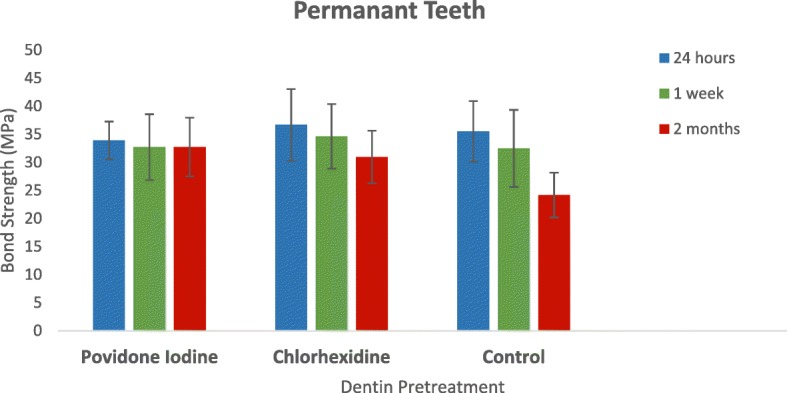
Means and Standard Deviations of the Micro-tensile bond strengths of the different groups at different storage periods in permanent teeth, Mega pascal (MPa). Within povidone iodine group (F = 0.746; *p* = 0.477), within chlorhexidine group and control group (F = 9.482, *p* < 0.001 and F = 46.036, *p* < 0.001, respectively)

A total of 376 specimens were subjected to MTBS testing and fracture mode analysis. There were 38 cohesive failures among the 376 sticks. These cohesive failures were either in dentin or in resin material, which does not represent the actual resin-dentin bond strength. Therefore, it was not included in the statistical analysis [12]. The remaining 338 specimens were included in the statistical analysis. The power of the sample size at an alpha value of 0.05 was 1.00. Figure 2 present the mean and standard deviation of each group.

One-way analysis of variance revealed that there was no significant reduction in resin-dentin bond strength in the PVP-I surface pretreatment group at any of the three storage time-points (F = 0.746; *p* = 0.477). Conversely, there were significant reduction in resin-dentin bond strength in the chlorhexidine surface pretreatment group and the control group (no pretreatment) at the three storage time-points (F = 9.482, *p* <  0.001 and F = 46.036, *p* < 0.001, respectively). The post-hoc Bonferroni test indicated that there was no significant loss of resin-dentin bond strength between 24 h and 1 week in the chlorhexidine group. In the control group, the mean bond strength at 1 week was significantly lower than that at 24 h. Additionally, in both the chlorhexidine group and the control group the mean bond strength at 2 months was significantly lower than those at 1 week and at 24 h. Detailed results from the post-hoc Bonferroni test are shown in Tables 1 and 2.

**Table 1:** Post-hoc test for resin-dentin micro-tensile bond strength in the chlorhexidine group

Post-hoc^a^		*p* value
24 h	1 week	0.386
1 week	2 months	0.021^*^
2 months	24 h	< 0.001^*^

^a^Bonferroni test

^*^Statistically significant at *p* < 0.05

**Table 2:** Post-hoc test for resin-dentin micro-tensile bond strength in the control group

Post-hoc^a^		*p* value
24 h	1 week	0.049*
1 week	2 months	< 0.001*
2 months	24 h	< 0.001*

^a^Bonferroni Test

^*^Statistically significant at *p* < 0.05

The analyses revealed significant associations between storage time and failure mode within the PVP-I group and the chlorhexidine group. Table 3 shows the associations between storage time and failure mode within each study group.

**Table 3:** Associations between storage time and failure mode in permanent teeth in each group

Dentin surface pretreatment	Type of failure	Storage time			Total (%)	*p* value^+^
		24 h (%)	1 week (%)	2 months (%)		
Povidone-iodine	Adhesive failure	38 (88.4)	32 (82.1)	26 (60.5)	96 (76.8)	0.007^*^
	Cohesive in dentin	0 (0)	0 (0)	4 (9.3)	4 (3.2)	
	Cohesive in composite	0 (0)	4 (10.3)	5 (11.6)	9 (7.2)	
	Mixed failure	5 (11.6)	3 (7.7)	8 (18.6)	16 (12.8)	
	Total	43 (100)	39 (100)	43 (100)	125 (100)	
Chlorhexidine	Adhesive failure	36 (90.0)	33 (84.6)	24 (61.5)	93 (78.8)	0.013^*^
	Cohesive in dentin	0 (0)	1 (2.6)	1 (2.6)	2 (1.7)	
	Cohesive in composite	2 (5.0)	0 (0)	5 (12.8)	7 (5.9)	
	Mixed failure	2 (5.0)	5 (12.8)	9 (23.1)	16 (13.6)	
	Total	40 (100)	39 (100)	39 (100)	118 (100)	
Control	Adhesive failure	37 (84.1)	36 (81.8)	30 (66.7)	103 (77.4)	0.053
	Cohesive in dentin	3 (6.8)	1 (2.3)	0 (0)	4 (3.0)	
	Cohesive in composite	1 (2.3)	3 (6.8)	8 (17.8)	12 (9.0)	
	Mixed failure	3 (6.8)	4 (9.1)	7 (15.6)	14 (10.5)	
	Total	44 (100)	44 (100)	45 (100)	133 (100)	
Total	Adhesive failure	111 (87.4)	101 (82.8)	80 (63.0)	292 (77.7)	< 0.001^*^
	Cohesive in dentin	3 (2.4)	2 (1.6)	5 (3.9)	10 (2.7)	
	Cohesive in composite	3 (2.4)	7 (5.7)	18 (14.2)	28 (7.4)	
	Mixed failure	10 (7.9)	12 (9.8)	24 (18.9)	46 (12.2)	
	Total	127 (100)	122 (100)	127 (100)	376 (100)	

^+^Fisher’s exact test

^*^Statistically significant at *p* < 0.05

## Discussion

In the present study, only freshly extracted, non-carious, permanent molars were utilized in an attempt to standardize the dentin substrates used. Intact resin-bonded teeth were stored in distilled water under simulated pulpal pressure. The water exposure of intact resin-bonded teeth may resemble a more realistic clinical situation in terms of hydrolytic degradation than smaller resin-dentin specimens directly exposed to water. Simulated pulpal pressure was used in this in vitro study in an attempt to achieve reliable results that were relevant to real clinical conditions. Simulated pulp pressures ranging from 30 to 37 cm H_2_O have been used in many studies investigating the influence of intra-pulpal pressure on the bonding effectiveness of adhesives to dentin [13–15]. In the current study, a lower intra-pulpal pressure was utilized because the intra-pulpal pressure in normal pulp is not as high as previously established [16]. Previous in vivo studies have reported that values of approximately 15 cm H_2_O should be used to simulate normal pulp pressure [16, 17].

In the current study, conventional two step etch-and-rinse adhesive was used (Single Bond, 3 M ESPE, St. Paul, MN, USA) which has a pH of 3.6 [18]. Previous studies have suggested that low pH (4.5) acids are capable of activating MMPs [4, 19]. Therefore, it was hypothesized that this adhesive would be capable of activating dentin proteolytic enzymes derived from the underlying partially-demineralized dentin [20].

In the current study, we used 10% PVP-I and 2% chlorhexidine as therapeutic primers of etched dentin. The PVP-I and chlorhexidine were applied to etched dentin before bonding, and the excess was air-dried without rinsing. The technique used in this experiment was a wet bonding technique. PVP-I and chlorhexidine digluconate are soluble in water. The differences in the binding performances of PVP-I and chlorhexidine to collagen and hydroxyl apatite are unknown.

Chlorhexidine has a high affinity to dentin. It can bind electrostatically to the phosphate groups of dentin and to carboxyl groups of collagen fibers. However, chlorhexidine composed of a large water-soluble molecule that might be leaded out of dentin over time [21]. Moreover, the binding mechanism between the PVP-I and the dentinal structure is not clear. However, the application of a miscible (capable of mixing in any ratio without separation of two phases) solution to water-saturated dentin after etching and rinsing should maximize chlorhexidine concentration within the hybrid layer [21]. Without rinsing, excess PVP-I and chlorhexidine may be incorporated into the primer, and released slowly over time [21]. Previous studies have reported that PVP-I has the capacity to be slowly released over time [22–24].

In the present study, the PVP-I group showed no significant reduction in dentin bond strength at the 24 h, 1 week, or 2 months time-points. In contrast, the chlorhexidine and control groups showed significant reductions in dentin bond strength at 1 week and at 2 months. The differences in mean dentin bond strength in the chlorhexidine and control groups between 24 h and 2 months were approximately 6 MPa and 11 MPa, respectively, while the corresponding reduction in the PVP-I group was only ~ 1 MPa. This finding may be explained by the superior water solubility of PVP-I compared to chlorhexidine, and the capacity of PVP-I to slowly release iodine over time [25], which ensures the establishment of a nontoxic, optimal concentration of iodine [26, 27]. This may have resulted in better penetration of PVP-I and inhibition of the MMPs within dentin.

In accordance with the results of previous studies [28–32], in the current study chlorhexidine application after acid etching had no effect on immediate or 1-week resin-dentin bond strength. This is concordant with previous in vitro [33] and in vivo [34] studies using an etch-and-rinse adhesive. Carrilho et al. [33] [34] found that treating etched dentin surfaces of permanent teeth with 2% chlorhexidine did not affect the in vitro or in vivo MTBS of specimens tested at 24 h. Furthermore, a meta-analysis of the effects of 2% chlorhexidine vs. control at baseline (immediate bond strength) revealed no statistically significant difference between groups [35].

In the current study, all the teeth were subjected to pulpal pressure simulation. Campos et al. [36] studied the effects of 0.2% and 2.0% chlorhexidine on dentin bonding durability. Two-step etch-and-rinse (Single-Bond) and all-in-one self-etch adhesive (Clearfil Tri S Bond) were used, and all the teeth were subjected to 30 cm H_2_O pulpal pressure and thermo-mechanical stressing. They reported that MTBS was significantly higher in the groups treated with two-step etch-and-rinse adhesive associated with 0.2% and 2.0% chlorhexidine than it was in the control group without chlorhexidine. Additionally, there were no significant differences in MTBS between the group treated with Clearfil Tri S Bond, the control group, and the group treated with 0.2% chlorhexidine after 6 months. Chlorhexidine was reportedly able to reduce the loss of bond strength of single bond adhesive, but not all-in-one self-etch adhesive, after storage for 6 months under simulated pulpal pressure. Those results are concordant with the results of the current study.

The current study had some limitations. The etch-and-rinse system was used in the current study to bond to sound dentin, and thus it may be that the hybrid layer in the current study was infiltrated by the water [37] utilized to exert the pulpal pressure on the dentin surface. However, the presence of an enamel rim sealing the boundaries of the specimens prevented the leaching out of any plasticized adhesive monomer into the storage media [36], and thus diminished the effect of pulpal pressure on the observed bond strength. Additionally, being an in vitro study the results do not directly reflect the clinical conditions of actual teeth, however, simulated pulp pressure was used during bonding and ageing to simulate in vivo conditions. Further clinical studies are needed to confirm the results of the current study.

To the best of our knowledge, this is the first report of data on the effects of PVP-I as a MMP inhibitor on the preservation of dentin bond durability. The results showed that PVP-I, an experimental therapeutic primer, also prevented bond strength deterioration over 2 months of aging. Further studies are needed to explore the reasons behind the preservation of the bond strength. Additionally, long-term studies are needed to evaluate the effects of PVP-I on bond strength. Further studies are also needed to study the inhibitory effects of PVP-I on MMPs directly.

## Conclusion

Povidone iodine pretreatment of etched dentin was effective in reducing the loss of bond strength over time, while the chlorhexidine pretreatment and negative control showed significant deterioration in micro-tensile bond strength over time in permanent teeth.

## Abbreviations

MMPs: Matrix metalloproteinases
MTBS: Micro-tensile bond strength
PVP-I: Povidone-iodine
